# Association between early childhood caries and diet quality among Chinese children aged 2–5 years

**DOI:** 10.3389/fpubh.2022.974419

**Published:** 2022-09-06

**Authors:** Xinfeng Wang, Zhe Ma, Min Lei, Caiyun Zhao, Xiuyan Lin, Fengdi Cao, Hong Shi

**Affiliations:** ^1^Department of Pediatric Dentistry, Hospital of Stomatology and Hebei Provincial Key Laboratory of Stomatology, Hebei Medical University, Shijiazhuang, China; ^2^Department of Preventive Dentistry, Hospital of Stomatology and Hebei Provincial Key Laboratory of Stomatology, Hebei Medical University, Shijiazhuang, China; ^3^Department of Nutrition, Third Hospital of Hebei Medical University, Shijiazhuang, China; ^4^Faculty of Dentistry, Melbourne University, Melbourne, VIC, Australia

**Keywords:** early childhood caries, diet quality, Chinese diet balance index for preschool children, Chinese children aged 2–5 years, dietary imbalance

## Abstract

**Background:**

Early childhood caries (ECC) is a major oral problem affecting the health and wellbeing of children worldwide. Diet quality is a better predictor of ECC risk than single foods or specific nutrients. The purposes of this study were to assess the associations between ECC and diet quality among 2- to 5-year-old Chinese children.

**Methods:**

A total of 150 eligible children were included in this study. The decayed, missing, or filled surface (dmfs) score was recorded for each child by dental examination. All participants were divided into three groups based on their age and dmfs score [the caries-free group, the ECC group, and the severe early childhood caries (S-ECC) group]. Parents were invited to complete a questionnaire on the general characteristics and oral health behaviors of the participants. The information of 24-h dietary intake from each child was captured *via* a mobile APP. The Chinese diet balance index for preschool children (DBI_C) indicators score, high bound score (HBS), low bound score (LBS), and diet quality distance (DQD) score were calculated to assess the diet quality of study subjects. The associations of ECC with HBS, LBS, DQD score, and DBI_C indicators score were analyzed by Mann-Whitney U test and multivariable logistic regression analysis.

**Results:**

In this study, 21, 31, and 98 children were diagnosed with caries-free, ECC, and S-ECC, respectively. Statistical analysis revealed that the risk of ECC and S-ECC were significantly increased with the DQD score (OR = 1.283 and 1.287, respectively), but both were not associated with HBS and LBS (*P* > 0.05). In the meantime, the risk of ECC and S-ECC were significantly increased with the Grains score (OR = 1.623 and 1.777, respectively), and significantly decreased with the Food diversity score (OR = 0.271 and 0.315, respectively). Moreover, the risk of S-ECC also significantly decreased with the Vegetables score (OR = 0.137).

**Conclusion:**

Both ECC and S-ECC were associated with a high degree of dietary imbalance and grains intake as well as a low degree of food diversity among Chinese children aged 2–5 years. In addition, S-ECC was also associated with a low degree of vegetable intake.

## Introduction

Early childhood caries (ECC), one of the most widespread chronic diseases, has posed a threat to children's health worldwide. It is defined by the American Academy of Pediatric Dentistry as a child under 71 months of age with one or more decayed (with or without cavitation lesions), missing (due to caries), or filled tooth surfaces (dmfs) in any primary tooth (1). The Global Burden of Disease Study 2016 indicated that the incidence rate of deciduous tooth caries ranked fifth among the most prevalent diseases in the world (2). The Fourth National Oral Health Epidemiological Survey Report 2018 demonstrated that 50.8, 63.6, and 71.9% of Chinese children aged 3, 4, and 5 experienced ECC, respectively, while the therapeutic rates were only 1.5, 2.9, and 4.1%, respectively (3). If not addressed, ECC can not only affect children's growth, wellbeing, and quality of life (4) but can also have a negative impact on parents and socio-economics (5). Therefore, it is a challenging issue faced by pediatric dentists worldwide to prevent ECC.

ECC is a multifactorial disease, with common risk factors including cariogenic microorganisms, inappropriate feeding practices, frequent contact with fermentable carbohydrates, poor oral hygiene habits, and a series of social variables (6, 7). It has been well recognized that diets play a critical role in the etiopathogenesis of ECC (8). Among various foods, sucrose and processed or hydrolyzed starchy foods are considered to have a high cariogenic potential (9). All of these contain carbohydrates that can be fermented by cariogenic bacteria in the oral cavity, causing the saliva pH to drop to 5.5 or lower and thus facilitating the formation of caries (9). In parallel, other foods have been discussed for their anti-cariogenic effects, for instance, fresh vegetables and fruits, unrefined grains (whole grains), milk, and dairy products (10, 11).

Traditional nutritional epidemiology investigates the association between chronic diseases and diets through a single (or few) food or specific nutrient. However, the effects of and interaction with other potentially contributory groups on ECC may be missed out (8). Diet quality refers to the extent to which the types, quantities, and proportions of major foods and/or nutrients in the diet align with dietary guidelines or proven healthy dietary structures (12). In recent years, exploring the effects of overall diet quality on ECC has garnered attention from researchers due to it takes into account the complex synergistic effects between foods. At present, the tools utilized to assess the diet quality for children with ECC are primarily the Healthy Eating Index-2005 (HEI-2005) and its updated version, which are measures for assessing whether a group of foods conformity with the Dietary Guidelines for Americans (13–15). In 2015, Zaki et al. (16) claimed that Egyptian children aged 2–6 years with S-ECC had significantly lower HEI-2005 scores than children without caries. In 2017, Elif Inan-Eroglu et al. (17) reported that ECC patients accounted for a higher percentage of children with lower HEI-2010 scores. In 2020, Priyadarshini and Gurunathan (18) observed that Indian children with higher HEI-2005 scores were less prone to ECC. So far, HEI-2005 and its updated version have not been utilized to assess the diet quality among Chinese children aged 2–5 years. Considering the dietary habits vary across countries and regions, younger children differ from adults in recommended intake and nutritional requirements, in addition to having special physiological characteristics such as gradual growth in height and weight, limited chewing, and digestive ability (19, 20). The diet quality of subjects in the current research was assessed by adopting the Chinese diet balance index for preschool children (DBI_C), which consists of 10 food group indicators listed in the Balanced Diet Pagoda for Chinese Preschool Children, namely Grains, Vegetables, Fruits, Dairy, Beans, Animal foods, Cooking oil, Salt, Drinking water, and Food diversity (21).

Accurate description of foods and precise estimation of portion sizes are critical for assessing dietary intake. Traditional methods used in the past (e.g., multiple 24-h diets recall and food frequency questionnaires) are subject to inherent errors that could lead to inaccurate assessments, negatively impacting patients and research outcomes. Recently, along with the popularization of cameras and mobile phones, image-based methods integrating mobile device application technologies have been developed (22). All the foods consumed by the subject are captured as images *via* a mobile device and recorded as the primary sources of dietary intake, which follow the method for recording diet (22). Parents/guardians can supplement image details to reduce the food under-reporting and improve dietary assessments' accuracy compared to traditional assessment methods alone (23). Therefore, a mobile phone APP based on the image-based method was used to acquire 24-h dietary intake information of the study participants.

The present study was conducted to investigate the associations of ECC and S-ECC with diet quality among Chinese children aged 2–5 years. We hypothesized that the risk of ECC and S-ECC is not associated with diet quality among Chinese children aged 2–5 years.

## Materials and methods

### Study population

A cross-sectional, analytical study design was carried out. One hundred and fifty healthy children were chosen by a convenience sampling method from the Department of Pediatric Dentistry at the Dental Hospital, Hebei Medical University, China, and three urban kindergartens in Shijiazhuang, China.

The eligibility criteria included children aged 2–5 who live with their parents/legal guardians, presence of primary dentition only.

Exclusion criteria were as follows: (1) Children with energy intake below 450 kcal/d or above 2,800 kcal/d (21); (2) Children whose height and weight are not within the normal range of the Reference Standards for Growth and Development of Children under 7 years old in China; (3) Children with any mental or systemic disease that affects oral health examination or dietary intake; (4) Children who took any antibiotics 2 weeks prior to this research.

### Sample size estimation

The determination of sample size with the following assumptions: ECC prevalence of 62.5% was generated from The Fourth National Oral Health Epidemiological Survey Report 2018 (3). A margin of error was set to be 0.15 times the prevalence, which is 62.5% ^*^ 0.15 = 9%. Type I error = 5%. The confidence level was 95%. The required sample size calculated by PASS (Version 15) was 120. Assuming that the loss of follow-up was randomized, the loss of follow-up rate of 10% would require 134 cases. A total of 150 children were finally enrolled in the analysis.

### Ethical considerations

This cross-sectional study was approved by the Ethics Committee of Dental Hospital, Hebei Medical University, China (No. [2018]028) and conducted in accordance with the principles of the Declaration of Helsinki. Parents/legal guardians of all eligible participants were informed about the research purpose, the health benefits, and potential hazards before the study commenced. They all provided written informed consent. All the data in this study were used for scientific research only. In addition, participants suffering from ECC or other oral diseases were offered the necessary advice and treatment.

### Dental examinations

Dental examinations were carried out under field conditions by two trained and calibrated pediatric dentists. Duplicate clinical examinations were conducted to test the reliability of intra-examiner, with kappa values averaging 90 and 88% for the examiners themselves and between the two examiners, respectively. To ensure the accuracy of the examinations as much as possible, two caries diagnostic criteria have been employed: the International Caries Detection and Assessment System (ICDAS-II) (24) was used to assess early enamel caries without visible cavity formation. In the meantime, World Health Organization (WHO) criteria (25) were used to determine cavitated lesions in pits, fissures, and smooth surfaces.

For early enamel caries, after food residues and debris were removed, the decayed (with or without cavitation lesions), missing (due to caries), or filled surface (dmfs) score of children was determined through the visual examination using the sterile dental mirror and the portable air compressor under sufficient illumination. For cavitated lesions, the dmfs score was determined using the sterile dental mirror under sufficient illumination after cleaning and drying the teeth. And if necessary, a community periodontal index (CPI) probe was carried out to clean debris from the pits or fissures without significant axial force or excessive pressure. The 150 children were divided into three groups depending on their age and total dmfs score: the caries-free group consisted of 21 children (dmfs = 0), the ECC group consisted of 31 children [based on the definition by the American Academy of Pediatric Dentistry (1)], and the S-ECC group consisted of 98 children [based on the definition by the American Academy of Pediatric Dentistry (1)].

### Questionnaire survey

Acquisition of the general characteristics and oral health behaviors of the study subjects were conducted using a questionnaire survey, which included questions pertaining to social demographics, infant feeding practices, and oral hygiene habits (Appendix 1).

### Twenty-four hour dietary intake

The data on 24-h dietary intake (one workday when children eat regularly) were acquired using a mobile phone APP (Beijing Sihai Huachen Technology Co., Ltd.), which consists of the Children's Household Nutrition Management Micro-platform and Children's Nutrition Supervision Micro-platform. Firstly, twenty parents/legal guardians were randomly selected to test whether they could use this APP proficiently and correctly. According to the active feedback from the parents/legal guardians, no further explanation is deemed necessary for the use of this software.

The parents/legal guardians uploaded all the food consumed by children within 24 h (including snacks and beverages), in the form of images, onto the Children's Household Nutrition Management Micro-platform, in which they can also mark and supplement the information on food intake. The next day, the researchers acquired the details about food intake from the Children's Nutrition Supervision Micro-platform. All food items received were confirmed with the diet uploader by phone. We will request parents to make corrections or re-upload the food images if any errors are found at any time during the above period to ensure the accuracy and validity of the data obtained. In the meantime, if the parents uploaded diets as well as confirmed or corrected, we provided them with feedback on their child's dietary status as compensation. By calculating the score of each indicator in DBI_C, each child's dietary status was assessed and specific dietary recommendations were given.

### Assessment of diet quality

Each food item acquired in the 24-h diet was transposed into the corresponding DBI_C indicators, and the score of each indicator was calculated (Appendix 2). The values of DBI_C indicators were determined by referring to Dietary Guidelines for Chinese preschool children (20) and Balanced Diet Pagoda for Chinese Preschool Children. A score closer to 0 means the intake of this food group is closer to the recommended intake at the corresponding age group.

According to the DBI_C score calculation method, the high bound score (HBS), low bound score (LBS), and diet quality distance (DQD) score were calculated, respectively. For these three indicators, a score closer to 0 means the dietary status is better. HBS is the absolute value of the sum of the positive score of DBI_C indicators, which reflects the degree of excessive dietary intake. Its score ranges from 0 to 36: 0 means no excessive intake; 1–7 means appropriate intake; 8–14, 15–22, and 23–36 means low, moderate, and high excessive intake, respectively. LBS is the absolute value of the sum of the negative score of DBI_C indicators, which reflects the degree of insufficient dietary intake. Its score ranges from 0 to 72: 0 means no insufficient intake; 1–14 means appropriate intake; 15–29, 30–43, and 44–72 means low, moderate, and high insufficient intake, respectively. DQD is the sum of the absolute value of the DBI_C indicators score, which comprehensively reflects the problem of dietary imbalance. Its score ranges from 0 to 84: 0 means that there is neither dietary insufficient nor excessive intake in the diet; 1–17 means appropriate, and 18–34, 35–50, and 51–84 means low, moderate, and high dietary imbalance, respectively.

### Statistical analysis

The statistical description was presented as means and standard deviation (SD) for continuous variables conforming to normality, the median and quartile range for those with skewness, furthermore frequencies and percentages (%) for categorical variables.

Univariate analysis was conducted using appropriate tests (*t*-test, Pearson χ^2^, Fisher exact test, and Mann-Whitney *U*-test) to evaluate associations of social demographics, infant feeding practices, and oral hygiene habits to ECC and S-ECC. Mann-Whitney *U*-test was utilized to compare the DBI_C indicators score, HBS, LBS, and DQD score between the study groups since the data distribution did not conform to normality and (or) heterogeneity of variance. All statistical tests were two-tailed with a statistical significance level of *P* ≤ 0.05.

The above indicators that showed significant statistical differences (HBS, DQD score, Grains score, Vegetables score, and Food diversity score) were included in multivariable logistic regression models, that were constructed to assess the independent effect of these variables on ECC (Model 1) and S-ECC (Model 2). To understand whether potential confounders could affect OR, we adjusted for the following covariates: age and adult supervision of toothbrushing. HBS, DQD score, Grains score, Vegetables score, Food diversity score, and age were modeled as continuous variables. Adult supervision of toothbrushing was categorized as yes or no. The assumption of linearity between the continuous independent variables and the logit transformed values for the dependent variables were assessed by the Box-Tidwell test. Multicollinearity between independent variables was checked using the Variance Inflation Factor. The−2 log-likelihood ratio test was used to test the overall significance of the model. The goodness-of-fit of the models was assessed by the Hosmer-Lemeshow. *P*-value ≤ 0.05 was considered statistically significant.

The data were processed, analyzed, and plotted on SPSS (Version 26) and Graphpad Prism (Version 9).

## Results

### General information

A total of 150 subjects aged 2–5 years meeting the study criteria were enrolled in our study and completed the program. Twenty-one of these individuals did not present with caries, 31 children were diagnosed with ECC, and 98 children were diagnosed with S-ECC. The general characteristics and oral health behaviors of study participants with the presence or absence of ECC and S-ECC are listed in Table 1. Subjects with ECC (3.871 ± 0.152) and S-ECC (3.684 ± 0.087) had higher age than those without caries (3.191 ± 0.214) (*P* = 0.010 and 0.021, respectively). A higher percentage of caries-free children (81%) were supervised to brush their teeth compared to children with ECC (45.2%) and S-ECC (56.1%) (*P* = 0.010 and 0.035, respectively). These differences were significant between the caries-free and the ECC groups as well as between the caries-free and the S-ECC groups (*P* < 0.05).

**Table 1:** The general characteristics and oral health behaviors of study participants with the presence or absence of ECC and S-ECC.

**Variable or practice**	**Caries-free**	**ECC**	**χ^2^/*t*/*z***	**S-ECC**	**χ^2^/*t*/*z***
	***n* (%)**	***n* (%)**	***P*_1_*-*value**	***n* (%)**	***P*_2_*-*value**
Age (years, mean ± SD)	3.191 ± 0.214	3.871 ± 0.152	*t* = −2.668 0.010*	3.684 ± 0.087	*t* = −2.333 0.021*
**Gender**
Boy	11 (52.4)	13 (41.9)	χ^2^ = 0.550	47 (48.0)	χ^2^ = 0.135
Girl	10 (47.6)	18 (58.1)	0.458	51 (52.0)	0.713
**Father's education level**
Junior high school or below	2 (9.5)	3 (9.6)	*z* = −0.035	12 (12.3)	*z* = 1.476
High school	4 (19.1)	6 (19.4)	0.972	35 (35.7)	0.140
University or above	15 (71.4)	22 (71.0)		51 (52.0)	
**Mother's education level**
Junior high school or below	2 (9.5)	4 (12.9)	*z* = −0.209	16 (16.3)	*z* = −1.618
High school	3 (14.3)	4 (12.9)	0.834	27 (27.6)	0.106
University or above	16 (76.2)	23 (74.2)		55 (56.1)	
**Sweets consumption**
Less than once a day or never	5 (23.8)	4 (12.9)	*z* = −1.559	15 (15.3)	*z* = −1.754
Once a day	9 (42.9)	10 (32.3)	0.119	29 (29.6)	0.079
More than once a day	7 (33.3)	17 (54.8)		54 (55.1)	
**Wean from breastfeeding (months)**
<12	5 (23.8)	9 (29.0)	*z* = −0.286	19 (19.4)	*z* = −1.508
12–18	13 (61.9)	14 (45.2)	0.775	45 (45.9)	0.132
>18	3 (14.3)	8 (25.8)		34 (34.7)	
**Start toothbrushing (months)**
<12	8 (38.1)	9 (29.0)	*z* = −0.783	26 (26.5)	*z* = −1.092
12–24	8 (38.1)	12 (38.7)	0.433	40 (40.8)	0.275
>24	5 (23.8)	10 (32.3)		32 (32.7)	
**Adult supervision of toothbrushing**
No	4 (19.0)	17 (54.8)	χ^2^ = 6.661	43 (43.9)	χ^2^ = 4.462
Yes	17 (81.0)	14 (45.2)	0.010*	55 (56.1)	0.035*
**Use of fluoride supplements**
No	8 (38.1)	16 (51.6)	χ^2^ = 0.920	47 (48.0)	χ^2^ = 0.677
Yes	13 (69.1)	15 (48.4)	0.377	51 (52.0)	0.411

*P*_1_ The significance level for comparison of general characteristics and oral health behaviors between caries-free and ECC groups. *P*_2_ The significance level for comparison of general characteristics and oral health behaviors between caries-free and S-ECC groups.

ECC, early childhood caries; S-ECC, severe early childhood caries.

*Significantly different at *P* < 0.05.

### Distribution and comparison of scores

**Figure 1:**
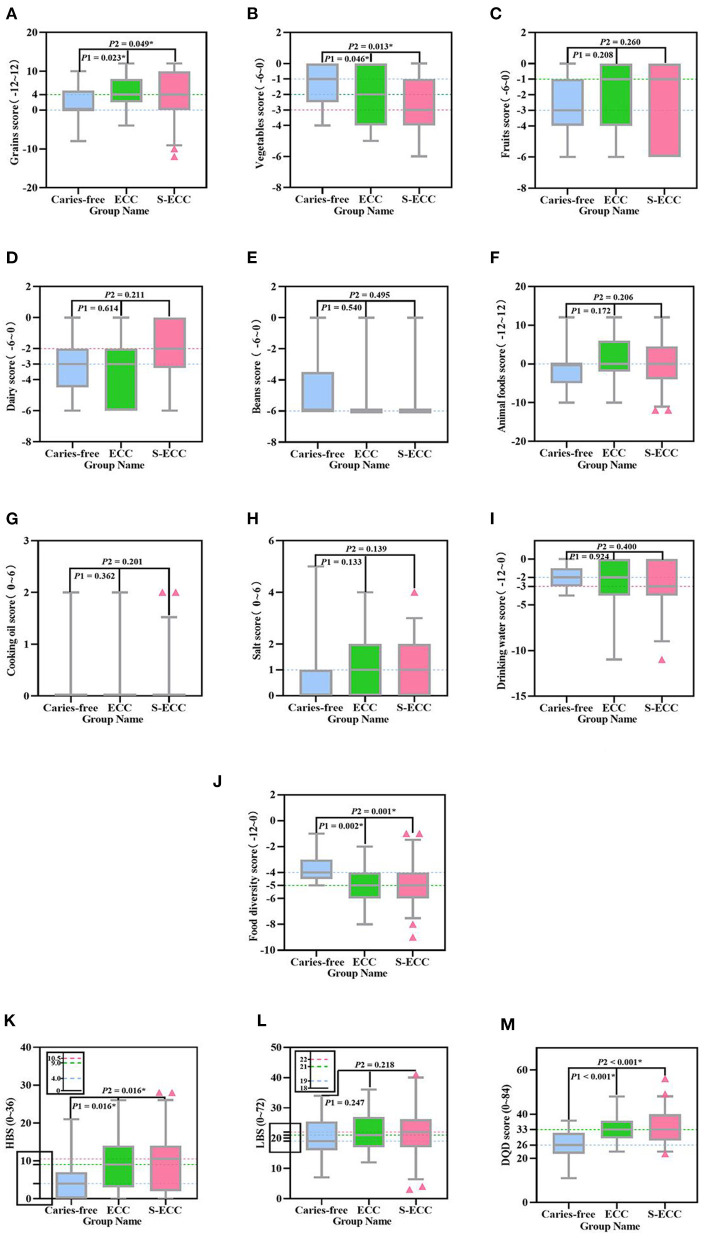
Distribution and comparison of DBI_C indicators scores, HBS, LBS, and DQD score among the three groups. The distribution and Comparison of **(A)** Grains score, **(B)** Vegetables score, **(C)** Fruits score, **(D)** Dairy score, **(E)** Beans score, **(F)** Animal foods score, **(G)** Cooking oil score, **(H)** Salt score, **(I)** Drinking water score, **(J)** Food diversity score, **(K)** HBS, **(L)** LBS, and **(M)** DQD score in the caries-free group, the ECC group, and the S-ECC group. The blue, green, and red dashed lines correspond to the median scores of 13 indicators in the caries-free group, the ECC group, and the S-ECC group, respectively, with the dashed lines overlapping for those with the same value. *P*1 Represents the significance level for the comparison of 13 indicators scores between the caries-free and the ECC groups. *P*2 Represents the significance level for the comparison of 13 indicators scores between the caries-free and the S-ECC groups. *Represents significant differences (*P* < 0.05) between the groups. The y-axis data corresponding to HBS and LBS are displayed by local enlarged images. HBS, high bound score; LBS, low bound score; DQD, diet quality distance.

The mean scores of Grains and Salt among the studied children were both higher than 0 (Figures 1A,H), while the mean scores of Vegetables, Fruits, Dairy, Beans, Drinking water, and Food diversity were all lower than 0 (Figures 1B–E,I,J), only the mean scores of Animal foods and Cooking oil were equal to 0 (Figures 1F,G). The distribution and comparison of DBI_C indicators score, HBS, LBS, and DQD score between the study groups are listed in Figure 1. Comparing the mean scores of 10 indicators in DBI_C, it was manifested that subjects with ECC or S-ECC had higher Grains score (*P* = 0.023 and 0.049, respectively) in addition to Vegetables score (*P* = 0.046 and 0.013, respectively) and Food diversity score (*P* = 0.002 and 0.001, respectively) that were lower than those without caries (Figures 1A,B,J). Other than that, subjects with ECC or S-ECC had higher HBS (the *P*-value for both was 0.016) and DQD score (the *P*-value for both was < 0.01) than did the subjects without caries (Figures 1K,M). These differences were significant between the caries-free and the ECC groups as well as between the caries-free and the S-ECC groups (*P* < 0.05).

### Multivariable logistic regression analysis for the associated factor of ECC and S-ECC

Indicators that showed statistically significant differences in univariate analysis (HBS, DQD score, Grains score, Vegetables score, and Food diversity score) were entered into the multivariable logistic regression analysis to assess their independent effects on ECC and S-ECC. Following adjustment for covariates (age, adult supervision of toothbrushing), ECC was associated with DQD score (*P* = 0.043), Grains score (*P* = 0.026), and Food diversity score (*P* = 0.015), and not associated with HBS (*P* = 0.432) and Vegetables score (*P* = 0.247) (Model 1 in Table 2). In parallel, S-ECC was associated with DQD score (*P* = 0.012), Grains score (*P* = 0.004), Vegetables score (*P* = 0.001), and Food diversity score (*P* = 0.011), and not associated with HBS (*P* = 0.569) (Model 2 in Table 2). On the other hand, the risk of ECC significantly increased with DQD score (OR = 1.283, 95% CI = 1.008-1.633) and Grains score (OR = 1.623, 95% CI = 1.060–2.483), whereas it was significantly decreased with Food diversity score (OR = 0.271, 95% CI = 0.095–0.779). A similar results was found for S-ECC, that its risk significantly increased with DQD score (OR = 1.287, 95% CI = 1.057–1.567) and Grains score (OR = 1.777, 95% CI = 1.195–2.641), whereas it was significantly decreased with Vegetables score (OR = 0.137, 95% CI = 0.042–0.451) and Food diversity score (OR = 0.315, 95% CI = 0.130–0.763).

**Table 2:** Multivariable logistic regression analysis for the associated factor of ECC and S-ECC.

**Variables**	**Model 1 (Outcome: ECC)**	**Model 2 (Outcome: S-ECC)**
	**OR (95% CI) *P-*value**	**OR (95% CI)** ***P*-value**
HBS	1.066 (0.909–1.249) 0.432	1.043 (0.903–1.205) 0.569
DQD score	1.283 (1.008–1.633) 0.043*	1.287 (1.057–1.567) 0.012*
Grains score	1.623 (1.060–2.483) 0.026*	1.777 (1.195–2.641) 0.004*
Vegetables score	0.699 (0.381–1.282) 0.247	0.137 (0.042–0.451) 0.001*
Food diversity score	0.271 (0.095–0.779) 0.015*	0.315 (0.130–0.763) 0.011*
Age	1.491 (0.325–6.846) 0.607	1.850 (0.347–9.867) 0.471
Adult supervision of toothbrushing	0.336 (0.025–4.525) 0.411	0.454 (0.017–11.923) 0.636

*Significantly different at *P* < 0.05.

ECC, early childhood caries; S-ECC, severe early childhood caries; OR, odds ratio; CI, confidence interval; HBS, high bound score; DQD, diet quality distance.

## Discussion

To the best of our knowledge, this current study provides the first evidence on the association of diet quality, as measured by the DBI_C to ECC among 2- to 5-year-old Chinese children. The indicators in DBI_C are all food groups and do not involve nutrients, which avoids the tedious calculation of nutrient-based dietary quality assessment methods, and allows for simpler and faster diet quality analysis for individuals and groups (21). In addition, the index adopts two-way scores, which can more intuitively reflect the problem and degree of dietary imbalance.

The main findings of this cross-sectional study were that those with ECC and S-ECC had a significantly higher degree of excessive dietary intake, dietary imbalance, and grains intake as well as a significantly lower degree of food diversity and vegetable intake than caries-free subjects. Multivariable logistic regression analysis revealed that the risk of ECC and S-ECC were significantly increased with the DQD score, but both were not associated with HBS and LBS. In the meantime, the risk of ECC and S-ECC were significantly increased with the Grains score and significantly decreased with the Food diversity score. Moreover, the risk of S-ECC also significantly decreased with the Vegetables score.

It is worth noting that the majority of participants in the present study had low-grade dietary imbalance, with both excessive and insufficient dietary intake. However, the mean scores of HBS, LBS, and DQD in the caries-free group were lower than those in the ECC group and the S-ECC group, which reflected that the caries-free children had higher diet quality and tended to follow healthy dietary recommendations. Similar results have been reported by Zaki et al. (16) and Priyadarshini et al. (18), both of whom found a significant association of a reduced likelihood of S-ECC with adherence to general healthy dietary guidelines among young children. The nutritional status of Chinese children has improved considerably with the rapid socio-economic development (26, 27). Nevertheless, it is still common for unbalanced dietary patterns among Chinese children (20, 28–30). In this study, only the mean intake of grains and salt for children aged 2–5 was higher than the recommended intake of Balanced Diet Pagoda for Chinese Preschool Children, while the mean intake of vegetables, fruits, dairy, beans, drinking water, and food diversity were below the recommended intake. The risk of ECC increased significantly with the degree of dietary imbalance. Dietary imbalances can lead to varying degrees of malnutrition, resulting in the hypofunction of salivary glands, altered salivary composition and reduced buffering capacity, and increased risk of ECC (31, 32). The level of diet quality in young children will affect the diet quality in school-age and even into adulthood (33) and is associated with the morbidity and mortality of chronic diseases from adulthood to old age (34, 35). Therefore, the problem of dietary imbalance in young children deserves great attention globally. Necessary intervention measures should be taken, such as strengthening nutrition and health awareness for guardians (36) and conducting interactive workshops for teachers and parents on nutrition education (37).

The mean scores of Grains were higher in children with ECC or S-ECC compared with those who did not present with caries. And the logistic regression models revealed that an increase in grains intake increased the probability of suffering from caries. It's a pity that DBI_C indicators do not strictly differentiate between refined grains and whole grains. However, grains consumption for Chinese residents was dominated by refined rice and flour as well as the intake of whole grains and coarse cereals were insufficient, as stated by the Scientific Research Report on Chinese Dietary Guidelines (38), which may indirectly indicate that the higher proportion of refined grains consumed compared to whole grains among Chinese children aged 2–5 years. Refined grains are lower in nutrients and minerals, dietary fiber content, polyunsaturated fatty acids, and phytochemicals, to a great extent attributable to loss of the outer bran layer and the endosperm of the grain being pounded during the most common way of refining (39), and starch accounts for the most proportion. Hancock et al. (40) claimed that the total time that plaque pH remained below a critical level of 5.5 when exposed to processed starch and sugar-containing foods was significantly higher compared to foods containing high concentrations of sucrose only, suggesting that an increased risk of caries was associated with the consumption of refined grains, particularly containing sucrose.

Moreover, this research which was conveying a significant difference in mean Vegetables score between the caries-free and ECC groups and between the caries-free and S-ECC groups, but logistic regression models showed it was an independent protective factor for S-ECC instead of ECC, indicating that S-ECC was significantly affected by vegetables intake with a stronger preventive effect than affected ECC. Vegetables are regarded to be of a protective role against caries for their fibrous nature and self-cleaning effects. Likewise, it stimulates saliva flow while chewing and meanwhile increases its acid-neutralizing power, which helps cleanse fermentable carbohydrates in the oral cavity (41, 42).

Children present with ECC or S-ECC had a higher mean Fruits score than children without caries. Still, the difference did not show statistical significance, which was inconsistent with the findings found by Zaki et al. (16), who concluded that caries experience was negatively associated with fruit intake. However, a few fruits, such as citrus fruits, are rich in organic acids (e.g., citric acids, malic acids, oxalic acids, and tartaric acids), which can decrease the saliva pH in the oral cavity, and excessive intake of such fruits may induce dental erosion and demineralization (11). In addition, the seasonal differences in the intake of fruits may also have an impact on the results.

Compared to the other two groups, children in the S-ECC group manifested the highest dairy intake, as demonstrated by their highest score, but the difference was not statistically significant. Although several studies have confirmed that milk and dairy products are abundant in nutrients, including proteins (e.g., casein and whey protein), minerals (e.g., calcium and phosphorus), and lipids (e.g., essential fatty acids and non-essential fatty acids) (43, 44), and yogurt and cheese also contain casein phosphopeptides (45, 46). *In vitro* demineralization/remineralization experiments also tend to indicate the low cariogenicity and potential caries-protective roles of milk (43). However, a high percentage of commercial milk and dairy products contain sucrose and flavoring to upgrade and enrich the odor and taste of the products, which complicates the interpretation of research into cariogenicity. Moreover, yogurt and cheese are relatively viscous foods, which can provide a substrate for bacteria and may raise a higher risk of caries when the products containing more sucrose or flavoring are consumed without cleaning the teeth promptly.

In this study, children who did not present with caries had the highest Food diversity score than children diagnosed with ECC and S-ECC. Meanwhile, logistic regression models revealed that children with a higher degree of food diversity were less likely to suffer from caries. Food diversity has been confirmed to be positively related to dietary micronutrient intake (29), and it was considered an indicator of great value for the prediction of macronutrient or micronutrient adequacy in children (30, 47). However, If a child has a poorly balanced diet, deficiencies in calcium, iron, albumin, vitamin D, and protein-energy malnutrition may induce enamel hypoplasia/hypomineralization, roughening the enamel surface and prone to plaque accumulation, with can lead to post-eruptive caries (32, 48–50). A balanced diet with various foods is quite essential for young children as most micronutrients are derived from the daily diet.

Image-based methods offer a more comprehensive range of viable options for dietary assessment, which is easier to incorporate into participants' daily lives. Furthermore, it can instantly record the diet consumed by an individual without relying on memory for input, which has the advantage of being fast, more efficient, and more precise compared to traditional methods. Several meaningful and valuable results were obtained, but some limitations should be noted. First and foremost, in this cross-sectional study, the findings only can reflect associations and cannot establish a causal relationship. Secondly, the limited sample size included in this study is due to time factors. Thirdly, DBI_C is a scale based on food group indicators and cannot assess the specific nutrient intake of children and thereby cannot analyze the association between ECC and nutrients. Finally, the image-based method relies on images to assess food intake. And the complex and diverse food cultures make the Chinese diet one of the most complex dietary systems worldwide. In the case of some unlisted food varieties, it cannot be identified or analyzed.

## Conclusion

Dietary imbalance is a serious health problem faced by this study sample. Both ECC and S-ECC were associated with a high degree of dietary imbalance and grains intake as well as a low degree of food diversity among Chinese children aged 2–5 years. Moreover, S-ECC was also associated with a low degree of vegetable intake. Nevertheless, large-scale prospective research is needed to validate these findings and to offer more information about the underlying causality and mechanism.

## Data availability statement

The raw data supporting the conclusions of this article will be made available by the authors, without undue reservation.

## Ethics statement

The studies involving human participants were reviewed and approved by Ethics Committee of Dental Hospital, Hebei Medical University, in Shijiazhuang, China (Ethics approval code: [2018]028). Written informed consent to participate in this study was provided by the participants' legal guardian/next of kin.

## Author contributions

HS conceived and designed the study. ZM, XL, ML, and CZ trained the data collectors. XW, ML, ZM, and CZ analyzed and reconciled the data. XW and FC drafted the first draft of the manuscript. HS reviewed and modified it to the final version. All authors contributed to manuscript revision, read, and approved the submitted version.

## Funding

This research was supported by the Hebei Provincial Department of Finance: Government-funded specialty competence Building and Professional leader Training (Professional leader) (Grant No: 0300000062), the Project funded by the Hebei Provincial Department of Finance: Study on the Correlation of Caries Activity and Dietary Nutrition among Younger Children (Grant No: 361029). The funders did not influence any stage of this study.

## Conflict of interest

The authors declare that the research was conducted in the absence of any commercial or financial relationships that could be construed as a potential conflict of interest.

## Publisher's note

All claims expressed in this article are solely those of the authors and do not necessarily represent those of their affiliated organizations, or those of the publisher, the editors and the reviewers. Any product that may be evaluated in this article, or claim that may be made by its manufacturer, is not guaranteed or endorsed by the publisher.

## References

[1] Drury TF Horowitz AM Ismail AI Maertens MP Rozier RG Selwitz RH. Diagnosing and reporting early childhood caries for research purposes. A report of a workshop sponsored by the National Institute of Dental and Craniofacial Research, the Health Resources and Services Administration, and the Health Care Financing Administration. J Public Health Dent. (1999) 59:192–7. 10.1111/j.1752-7325.1999.tb03268.x 10649591

[2] GBD 2016 Disease and Injury Incidence and Prevalence Collaborators. Global, regional, and national incidence, prevalence, and years lived with disability for 328 diseases and injuries for 195 countries, 1990-2016: a systematic analysis for the Global Burden of Disease Study 2016. Lancet. (2017) 390:1211–59. 10.1016/S0140-6736(17)32154-2 28919117 PMC5605509

[3] Wang X. The Fourth National Oral Health Epidemiological Survey Report. Beijing: People's Medical Publishing House (2018). p. 228.

[4] Ozsin OC Cocco P Cakir B. Dental caries and quality of life among preschool children: a hospital-based nested case-control study. Br Dent J. (2020) Online ahead of print. 10.1038/s41415-020-2317-9 33244147

[5] Rajab LD. Abdullah RB. Impact of dental caries on the quality of life of preschool children and families in Amman, Jordan Oral Health. Prev Dent. (2020) 18:571–82. 10.3290/j.ohpd.a44694 32515430 PMC11654531

[6] Anil S Anand PS. Early childhood caries: prevalence, risk factors, and prevention. Front Pediatr. (2017) 5:157. 10.3389/fped.2017.00157 28770188 PMC5514393

[7] Kirthiga M Murugan M Saikia A Kirubakaran R. Risk factors for early childhood caries: a systematic review and meta-analysis of case control and cohort studies. Pediatr Dent. (2019) 41:95–112. 30992106 PMC7100045

[8] Hu S Sim YF Toh JY Saw SM Godfrey KM Chong YS. Infant dietary patterns and early childhood caries in a multi-ethnic Asian cohort. Sci Rep. (2019) 9:852. 10.1038/s41598-018-37183-5 30696871 PMC6351619

[9] Berkowitz RJ. Causes treatment and prevention of early childhood caries: a microbiologic perspective. J Can Dent Assoc. (2003) 69:304–7. 12734024

[10] Nunn ME Braunstein NS Krall Kaye EA Dietrich T Garcia RI Henshaw MM. Healthy eating index is a predictor of early childhood caries. J Dent Res. (2009) 88:361–6. 10.1177/0022034509334043 19407158 PMC2774803

[11] Zeng X Tai B. Modern dietary patterns and oral health. Chin J Stomatol. (2020) 55:704–9. 10.3760/cma.j.cn112144-20200611-00332 33045779

[12] Brát J Vrablík M Herber O. Dietary changes in relationship to risk factors and coronary heart disease mortality. Vnitr Lek. (2015) 61:815–20. 26465281

[13] Guenther PM Reedy J Krebs-Smith SM Reeve BB. Evaluation of the healthy eating index-2005. J Am Diet Assoc. (2008) 108:1854–64. 10.1016/j.jada.2008.08.011 18954575

[14] Guenther PM Casavale KO Reedy J Kirkpatrick SI Hiza HAB Kuczynski KJ. Update of the healthy eating index: HEI-2010. J Acad Nutr Diet. (2013) 113:569–80. 10.1016/j.jand.2012.12.016 23415502 PMC3810369

[15] Reedy J Lerman JL Krebs-Smith SM Kirkpatrick SI Pannucci TE Wilson MM. Evaluation of the healthy eating index-2015. J Acad Nutr Diet. (2018) 118:1622–33. 10.1016/j.jand.2018.05.019 30146073 PMC6718954

[16] Zaki NAA Dowidar KML Abdelaziz WEE. Assessment of the Healthy Eating Index-2005 as a predictor of early childhood caries. Int J Paediatr Dent. (2015) 25:436–43. 10.1111/ipd.12150 25532620

[17] Inan-Eroglu E Özşin-Özler C Erçim RE Büyüktuncer Z Uzamiş-Tekçiçek M Güçiz-Dogan B. Is diet quality associated with early childhood caries in preschool children? A descriptive study. Turk J Pediatr. (2017) 59:537–47. 10.24953/turkjped.2017.05.006 29745115

[18] Priyadarshini P Gurunathan D. Role of diet in ECC affected South Indian children assessed by the HEI-2005: a pilot study. J Family Med Prim Care. (2020) 9:985–91. 10.4103/jfmpc.jfmpc_851_19 32318455 PMC7113943

[19] Chinese Nutrition Society. Chinese Dietary Reference Intakes (2013 Edition). Beijing: Science Press (2015). p. 660.

[20] Chinese Nutrition Society. Chinese Dietary Guidelines. Beijing: People's Medical Publishing House (2016). p. 343.

[21] Fang Y He Y Li C. Evaluation of dietary quality of Chinese preschool children based on Chinese diet balance index for preschool children. Chin J Prevent Med. (2020) 54:662–7. 10.3760/cma.j.cn112150-20190909-00719 32842283

[22] Boushey CJ Spoden M Zhu FM Delp EJ Kerr DA. New mobile methods for dietary assessment: review of image-assisted and image-based dietary assessment methods. Proc Nutr Soc. (2017) 76:283–94. 10.1017/S0029665116002913 27938425

[23] Höchsmann C Martin CK. Review of the validity and feasibility of image-assisted methods for dietary assessment. Int J Obes. (2020) 44:2358–71. 10.1038/s41366-020-00693-2 33033394 PMC7686022

[24] Dikmen B. Icdas II criteria (international caries detection and assessment system). J Istanb Univ Fac Dent. (2015) 49:63–72. 10.17096/jiufd.38691 28955548 PMC5573507

[25] World Health Organization. Oral Health Surveys-basic Methods Fifth Edition. Geneva: World Health Organization (2014). p. 132.

[26] Dearth-Wesley T Wang H Popkin BM. Under- and overnutrition dynamics in Chinese children and adults (1991-2004). Eur J Clin Nutr. (2008) 62:1302–7. 10.1038/sj.ejcn.1602853 17637598

[27] Tzioumis E Adair LS. Childhood dual burden of under- and overnutrition in low- and middle-income countries: a critical review. Food Nutr Bull. (2014) 35:230–43. 10.1177/156482651403500210 25076771 PMC4313560

[28] Wang H Wang D Ouyang Y Huang F Ding G Zhang B. Do Chinese children get enough micronutrients? Nutrients. (2017) 9:397. 10.3390/nu9040397 28420205 PMC5409736

[29] Meng L Wang Y Li T Loo-Bouwman CAV Zhang Y Man-Yau Szeto I. Dietary diversity and food variety in chinese children aged 3–17 years: are they negatively associated with dietary micronutrient inadequacy? Nutrients. (2018) 10:1674. 10.3390/nu10111674 30400573 PMC6267553

[30] Zhao W Yu K Tan S Zheng Y Zhao A Wang P. Dietary diversity scores: an indicator of micronutrient inadequacy instead of obesity for Chinese children. BMC Public Health. (2017) 17:440. 10.1186/s12889-017-4381-x 28499361 PMC5429576

[31] Folayan MO El Tantawi M Schroth RJ Vukovic A Kemoli A Gaffar B. Associations between early childhood caries, malnutrition and anemia: a global perspective. BMC Nutr. (2020) 6:16. 10.1186/s40795-020-00340-z 32467766 PMC7197144

[32] Psoter WJ Reid BC Katz RV. Malnutrition and dental caries: a review of the literature. Caries Res. (2005) 39:441–7. 10.1159/000088178 16251787 PMC1362953

[33] da Costa MP Durão C Lopes C Vilela S. Adherence to a healthy eating index from pre-school to school age and its associations with sociodemographic and early life factors. Br J Nutr. (2019) 122:220–30. 10.1017/S0007114519001028 31196225

[34] Batty GD Calvin CM Brett CE Cukić I Deary IJ. Childhood body weight in relation to morbidity from cardiovascular disease and cancer in older adulthood: 67-year follow-up of participants in the 1947 Scottish Mental Survey. Am J Epidemiol. (2015) 182:775–80. 10.1093/aje/kwv154 26443418

[35] Ness AR Maynard M Frankel S Smith GD Frobisher C Leary SD. Diet in childhood and adult cardiovascular and all cause mortality: the Boyd Orr cohort. Heart. (2005) 91:894–8. 10.1136/hrt.2004.043489 15958357 PMC1768996

[36] Durão C Severo M Oliveira A Moreira P Guerra A Barros H. Association of maternal characteristics and behaviours with 4-year-old children's dietary patterns. Matern Child Nutr. (2017) 13:e12278. 10.1111/mcn.12278 27040460 PMC6866190

[37] Vio F Salinas J Montenegro E González CG Lera L. Impact of a nutrition education intervention in teachers, preschool and basic school-age children in Valparaiso region in Chile. Nutr Hosp. (2014) 29:1298–304. 10.3305/nh.2014.29.6.7409 24972466

[38] China Nutrition Society. Scientific Research Report on Chinese Dietary Guidelines. Beijing: People's Medical Publishing House (2021). p. 192.

[39] Swaminathan S Dehghan M Raj JM Thomas T Rangarajan S Jenkins D. Associations of cereal grains intake with cardiovascular disease and mortality across 21 countries in Prospective Urban and Rural Epidemiology study: prospective cohort study. BMJ. (2021) 372:m4948. 10.1136/bmj.m4948 33536317 PMC7856570

[40] Hancock S Zinn C Schofield G Thornley S. Nutrition guidelines for dental care vs. the evidence: is there a disconnect? N Z Med J. (2020) 133:65–72. 32027640

[41] Yoshihara A Watanabe R Nishimuta M Hanada N Miyazaki H. The relationship between dietary intake and the number of teeth in elderly Japanese subjects. Gerodontology. (2005) 22:211–8. 10.1111/j.1741-2358.2005.00083.x 16329229

[42] Burt BA Eklund SA. Dentistry, Dental Practice, and the Community. Saint Louis, W. B. Saunders Company (2005). p. 440.

[43] Woodward M Rugg-Gunn AJ. Chapter 8: milk, yoghurts and dental caries. Monogr Oral Sci. (2020) 28:77–90. 10.1159/000455374 31940625

[44] Dror DK Allen LH. Dairy product intake in children and adolescents in developed countries: trends, nutritional contribution, and a review of association with health outcomes. Nutr Rev. (2014) 72:68–81. 10.1111/nure.12078 24330063

[45] Ferrazzano GF Cantile T Quarto M Ingenito A Chianese L Addeo F. Protective effect of yogurt extract on dental enamel demineralization *in vitro*. Aust Dent J. (2008) 53:314–9. 10.1111/j.1834-7819.2008.00072.x 19133946

[46] Kashket S DePaola DP. Cheese consumption and the development and progression of dental caries. Nutr Rev. (2002) 60:97–103. 10.1301/00296640260085822 12002685

[47] Nithya DJ Bhavani RV. Dietary diversity and its relationship with nutritional status among adolescents and adults in rural india. J Biosoc Sci. (2018) 50:397–413. 10.1017/S0021932017000463 28967344

[48] Mohamed WE Abou El Fadl RK Thabet RA Helmi M Kamal SH. Iron deficiency anaemia and early childhood caries: a cross-sectional study. Aust Dent J. (2021) 66(Suppl. 1):S27–36. 10.1111/adj.12842 33840096

[49] Atasoy HB Ulusoy ZI. The relationship between zinc deficiency and children's oral health. Pediatr Dent. (2012) 34:383–6. 23211913

[50] Olczak-Kowalczyk D Kaczmarek U Gozdowski D Turska-Szybka A. Association of parental-reported vitamin D supplementation with dental caries of 3-year-old children in Poland: a cross-sectional study. Clin Oral Investig. (2021) 25:6147–58. 10.1007/s00784-021-03914-8 33834312 PMC8531070

[51] Wang X Ma Z Lei M Zhao C Lin X Cao F. Correlations between diet quality and early childhood caries among 2- to 5-year-old Chinese children: a cross-sectional study. Research Square [Preprint] (2022). 10.21203/rs.3.rs-1503777/v1

