# Supplementary material for: Association between early childhood caries and diet quality among Chinese children aged 2–5 years
Source: Front Public Health. 2022 Sep 6;10:974419. doi: 10.3389/fpubh.2022.974419 (PMC9782538; doi:10.3389/fpubh.2022.974419)
Supplement: Supplementary file 1 [file Data_Sheet_1.PDF]

Questionnaire

Child's name: Gender: Date of Birth: Height: cm

Weight: kg Home Address:

Phone Number:

Q1: Which of the following is the educational level/highest education of the child's father?

A. Junior high school or below B. High School C. University or above

Q2: Which of the following is the educational level/highest education of the child's mother?

A. Junior high school or below B. High School C. University or above

Q3: How often does your child consume sweets?

A. Less than once a day or never B. Once a day C. More than once a day

Q4: How old was your child when weaning from breastfeeding?

A. <12 months B. 12-18 months C. >18 months

Q5: How old was your child when he/she started brushing? (includes parental assistance or supervision in brushing teeth)

A. <12 months B. 12-24 months C. >24 months

Q6: Do parents supervise the child to brush his/her teeth every day?

A. No B. Yes

Q7: Does your child use fluoride supplements (e.g., fluoride tablets and fluoride drops)?

A. No B. Yes
