# Supplementary material for: Association between early childhood caries and diet quality among Chinese children aged 2–5 years
Source: Front Public Health. 2022 Sep 6;10:974419. doi: 10.3389/fpubh.2022.974419 (PMC9782538; doi:10.3389/fpubh.2022.974419)
Supplement: Supplementary file 2 [file Data_Sheet_2.PDF]

Indicators of Chinese diet balance index for preschool children and their value method

Indicators	Score ranges	Children aged 2 and 3 years old	Children aged 4 and 5 years old
Grains[a]	-12~12	0g = -12, 75~125g = 0, >200g = 12; for every 15g increase (decrease), the score will increase (decrease) by 2 points	0g = -12, 100~150g = 0, >250g = 12; for every 20g increase (decrease), the score increases (decreases) by 2 points
Vegetables and Fruits[b]	-12~0		
Vegetables	-6~0	⩾100g = 0, 0g = -6; for every 20g decrease, the score decreases by 1 point	⩾150g = 0, 0g = -6; for every 30g decrease, the score decreases by 1 point
Fruits	-6 ~0	⩾100g = 0, 0g = -6; for every 20g decrease, the score decreases by 1 point	⩾150g = 0, 0g = -6; for every 30g decrease, the score decreases by 1 point
Dairy and Beans[c]	-12~0		
Dairy	-6~0	⩾350g = 0, 0g = -6; for every 70g decrease, the score decreases by 1 point	Same as 2 and 3 years old
Beans	-6~0	⩾5g = 0, 0g = -6; for every 1g decrease, the score decreases by 1 point	⩾10g = 0, 0g = -6; for every 2g decrease, the score decreases by 1 point
Animal foods[d]	-12~12	0g = -12, 75~125g = 0, >200g = 12; for every 15g increase (decrease), the score will increase (decrease) by 2 points	0g = -12, 100~150g = 0, >250g = 12; for every 20g increase (decrease), the score increases (decreases) by 2 points
Cooking oil	0~6	⩽20g = 0, >45g = 6; for every 5g increase, the score increases by 1 point	⩽25g = 0, >50g = 6; for every 5g increase, the score increases by 1 point
Salt	0~6	⩽2g = 0, >12g = 6; for every 2g increase, the score increases by 1 point	⩽3g = 0, >13g = 6; for every 2g increase, the score increases by 1 point
Drinking water	-12~0	⩾600ml = 0, <50ml = -12; for every 50ml decrease, the score decreases by 1 point	⩾700ml = 0, <40ml = -12; for every 60 ml decrease, the score decreases by 1 point
Food Diversitye	-12~0	The intake of all 12 sub-categories is 0g = -12, and the intake of all 12 sub-categories is more than 0g = 0; for each sub-category food intake of 0g, the score decreases by 1 point	Same as 2 and 3 year olds

Note:
[a] Grains include rice and its products, wheat and its products, dry beans (except soybeans) and root-tuber food, and the intake is based on the dry weight of raw food, sweet potato intake is divided by 3, potato by 4, and yam by 6. [b] Vegetables and fruits include fresh vegetables and fresh fruits, excluding processed products such as dried and preserved fruit. [c] Dairy includs milk, yogurt, and other kinds of dairy products. Beans include soybeans and other kinds of soy products. [d] Animal foods include livestock, poultry meat and their products, wild animal meat, fish, shrimp, aquatic products, and eggs. [e] Food diversity includes 12 sub-categories: rice and its products, wheat and its products, other grains and their products, dark vegetables, light colored vegetables, fruits, soybeans and their products, dairy and its products, livestock meat and its products, poultry meat and its products, eggs, fish, and shrimps.
